# Artificial Gramicidins

**DOI:** 10.3389/fchem.2019.00611

**Published:** 2019-09-04

**Authors:** Zhanhu Sun, Mihail Barboiu

**Affiliations:** Institut Europeen des Membranes, Adaptive Supramolecular Nanosystems Group, University of Montpellier, ENSCM-CNRS, Montpellier, France

**Keywords:** gramicidin A, biomimetic, ion channels, hydrophobic, hydrophilic

## Abstract

Gramicidin A, gA is a natural protein channel with a well-established, simple structure, and function: cations and water are transported together along the channel. Importantly, the dipolar orientation of water molecules within the pore can influence the ionic translocation. The need for simple artificial systems biomimicking the gA functions has been desired and they were until last decade unknown. Several interesting papers highlighted in this minireview have been published and supramolecular systems described here can be considered as primitive gA mimics. The dynamics of ions/water and protons confined within gA channels is difficult to structurally analyze and simpler artificial systems designed at the atomic level would have a crucial relevance for understanding such translocation scenarios at the molecular level. The directional ordering of confined water-wires or ions, as observed inside primitive gA channels is reminiscent with specific interactions between water and the natural gA. This dipolar orientation may induce specific dielectric properties which most probably influence the biological recognition at bio-interfaces or translocation of charge species along artificial channel pathways.

**Graphical Abstract:**
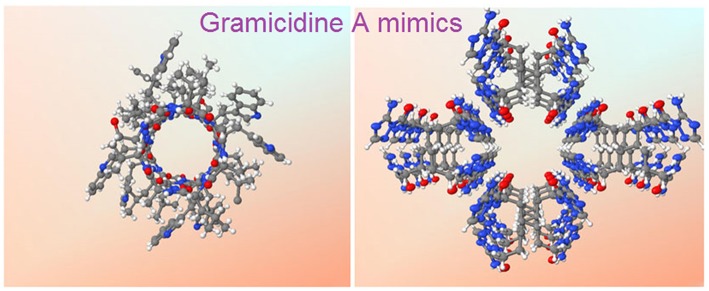
X-ray crystal structures of gA **(left)** and the hydrophilic T-channel of triazole 3 **(right)** at the same dimensional scale.

## Introduction

Gramicidin A, gA discovered during the late 30 s (Dubos, 1939), is one of the most studied natural channels (Burkhart et al., 1998; Roux, 2002; Allen et al., 2007) and important insights were obtained with synthetically modified gAs to improve their membrane transport activity (Pfeifer et al., 2006).

Concerning translocation mechanisms along gA pore, its polarized structure helps to compensate the high energy barrier to water and ion dehydration, which are transported sharing the unique pore pathway, through the bilayer membrane (Burkhart et al., 1998; Roux, 2002). Importantly, the dipolar orientation of water molecules within the pore can influence the ionic translocation and this process is also a determinant for their selective pumping in other protein-channels (Allen et al., 2007). Water, can influence, by its dynamic structure and orientation, ion and proton translocation and ion-valence selectivity of the gA channel.

The field of artificial ion channels have been extensively reviewed (Gokel and Mukhopadhyay, 2001; Sakai et al., 2005; Sakai and Matile, 2013). Understanding the dynamics of water molecules at the molecular level, hydrated ions, and protons within structurally simpler artificial channels would have a crucial relevance in order to understand many biological translocation processes involving dynamic transport through complicated protein channels (Barboiu, 2012; Barboiu and Gilles, 2013). The ability to know how ions or water-clusters are confined in structurally well-defined architectures might shed-light on the water structural behaviors within pores as observed with biological water (Kocsis et al., 2018), with properties at the boundaries between solid and liquid phases. Despite multiple studies of entrapping water or water/ion clusters within complex supramolecular structures, few synthetic channels have been tested to selectively transport water (Le Duc et al., 2011) and ions (Barboiu et al., 2014) efficiently through bilayer membranes.

Among the successful investigations for the construction of active artificial channels, one way is to use unimolecular channels (Hu et al., 2012). Another way is the bottom-up supramolecular strategy, in which biomimetic or bio-inspired artificial channel architectures are constructed *via* the self-assembly of synthetic molecular components through non-covalent self-assembly (Sakai et al., 2005; Cazacu et al., 2006; Ma et al., 2008).

Within this context, the need for simplest artificial systems biomimicking the complex natural gA functions has been desired and they were unknown until last 5 years. This Minireview will focus on recent accomplishments on artificial biomimetic ion channels, which can be envisioned as primitive gA mimics, presenting ion/proton, and water-channel conductance states in lipid bilayer membranes.

## Artificial gA Channels

Although gA is one of the most-simple and well-studied natural channels, important works in improving natural transport activity has been described in several findings, showing that gA can be bio-mimicked using artificial compounds with similar functions *like the natural gA*, in order to obtain *artificial gAs* by using simple compounds approaches. These systems are channel-type superstructures formed by self-assembly and provide remarkable combinations of functions similar to gA channel: water permeability, proton conductance via Grottus mechanism, cation vs. anion selectivity, single-channel activity. Within this context, the novel artificial systems may provide interesting information about the translocation mechanisms of water molecules or ions through channels within lipid bilayers. Meanwhile, their functions are close or comparable, even superior to the natural ion channel proteins.

Among the numerous investigations on ion channels, the bottom-up supramolecular biomimetic strategy uses simple synthetic molecules that self-assemble through non-covalent interactions: H- bonding, charge compensation or hydrophobic effects. Due to their easy manipulation, convenient modification and versatility, supramolecular biomimetic strategies are envisioned as an excellent method to further understand the functions, structures and mechanisms of ion channels, even to create substitutes for natural channels. Supramolecular chemists have concentrated their research on the study on artificial biomimetic structures of gA.

**Figure 1:**
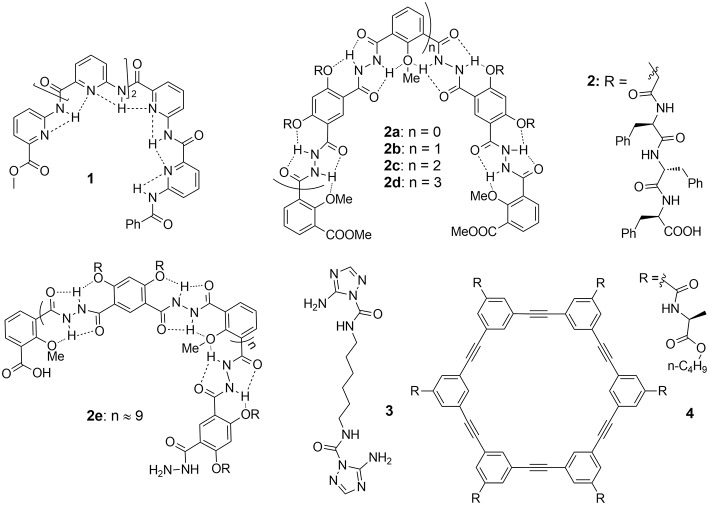
Artificial Gramicidin A ion channel forming compounds: aromatic foldameric polyamide **1**, aromatic foldameric hydrazides **2a**–**2e**. bola-amphiphile triazole **3** and arylene-ethynylene macrocycle **4**.

**Figure 2:**
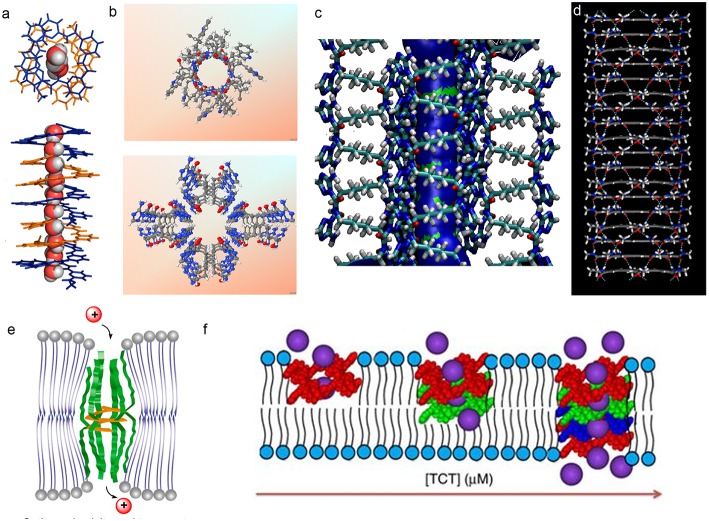
**(a)** Single crystal structure of **1** and the accompanying water wires; **(b)** X-ray crystal structures of gA (top) and the hydrophilic T-channel of triazole **3** (bottom) at the same dimensional scale, **(c)** Probability plots describing the average distribution of water molecules confined within the T-channels **(d)** simulated hydrophobic tubular pores from macrocycle **4**. Schematic representation on reconstitution of **(e) 2b** and **2c** and **(f) 3** in bilayer membrane models.

Zeng et al. prepared foldameric channels for synergetic transport of protons and of water (Zhao et al., 2014). They discovered that pentamer **1** (Figure 1) made from 6-aminopyridine-2-carboxylic components, which clearly shows the formation of a chiral helical structure, providing like in natural gA, a perfect pore dimensionally adapted (~2.8 Å) for water recognition. This structure is regarded as a model for the building of ion channels using a bottom-up self-assembling method. Interestingly, oriented water wires are oriented in one direction following the supramolecular chiral orientation of the helical molecules (Figure 2a). After unsuccessful tests on water transport under salt-induced osmotic conditions, the helical channels can effectively transport water only when a proton gradient is applied. Using the dynamic light scattering, the size of LUVs containing the pentamer **1** rapidly increased 40% within the first 15 min, in comparison to the inactivity of gA under the same conditions. The authors define this behavior as “proton gradient-induced water transport.” More interestingly, the pentamer **1** facilitates transmembrane proton transport, which is efficient and very similar to that of gA.

Li et al. have constructed hydrogen bonding-mediated hydrazide foldamers and have focused on building artificial biomimetic structures and responsive materials based on hydrogen bonding-mediated hydrazides and amides (Zhang et al., 2014). They have conceived and carried out a series of unimolecular channels (**2a**–**e**, Figure 1), whose selectivities and permeabilies even outmatch the natural gA. The structural stabilization in the lipid bilayers results from the multiple intramolecular hydrogen bonding, which direct half of the carbonyl groups toward the internal cavity, similar to gA and from the phenylalanine tripeptides acting as the lipophilic anchors. Patch clamp experiments show that the selectivities toward alkali ions correspond to the energetic penalty for ions dehydration. The helical **2d** and **2e** show higher ${\text{NH}}_{4}^{+}$/K^+^ selectivity than gA, under the identical performed conditions. The authors attribute this feature to the addition hydrogen bonding between ammonium cations and the hydrazide carbonyl moieties, which is supported by the increasing transporting ability toward ammonium with the elongation of the channel-forming compounds. In addition, stopped-flow experiments point out that these hydrazides display positive transporting activities toward Tl^+^ ions and function as a unimolecular channel process. Interestingly, the short helical **2b** transports Tl^+^ almost as effectively as gramicidin A does (Xin et al., 2014). According to stopped-flow experiments for the transport of Tl^+^, compounds **2b** and **2c** most probably formed a unimolecular channel in lipid bilayers (Figure 2e).

Barboiu et al. have described one of the most appropriate primitive artificial gA channels, showing amazing similarities in both structure and function aspects (Barboiu et al., 2014). The synthesis of a bola-amphiphile compound **3** (Figure 1) is the result of serendipity. It results in the formation of T-channels with a water filled interior free channel of 5 Å in van der Waals diameter, which is dimensionally similar to the gA channel (Figures 2b,c). The chiral T-channel is regarded as hydrophilic, because the carbonyl groups directing the channel inward toward the transport void, are in close contact with water like the carbonyl strings in gA. The total dipolar orientation of water molecules within the chiral pore determine the translocation of both protons and ions, that diffuse along such hydrophilic directional pathways. From a functional aspect, compound **3** is able to form transmembrane channels to facilitate water permeability and selective ion/proton transporting as well as single channel ion transporting. Based on molecular simulations and the transport data, compound **3** was likely to form T-channels in lipid bilayers (Figure 2e). Dynamic light scattering measurements prove that **3** effectively transports water. Compound **3** shows fifty times higher water permeability than the control. In addition, compound **3** constructs channels to transport protons via the inside water wires through the Grothuss mechanism. Furthermore, both cations and anions are effectively transmembrane moved, thus good cation/anion selectivity is accomplished. Theoretical simulations and experimental assays reveal that the conduction through the T-channel, like in gA, presents proton/water conduction, cation/anion selectivity, and large open channel-conductance states. The strong interactions between water molecules and the groups at the inner surface of the T-channel groups determine a net-dipole orientation of confined water molecules. Moreover, even when different ions are present within the channel, confined water remains significantly localized and still presents directional orientation and ordering within the T-channel (Barboiu et al., 2015). The T-channels—associating supramolecular chirality and dipolar water orientation—represent an interesting artificial mimic of gA.

Hydrophobic effects play an important role in biology, such as they are a significant driving force controlling the protein folding or facilitating the transmembrane ion and water transport. Within this context, Gong et al. have constructed a series of tubular hydrophobic adjustable nanopores (Figure 2D) generated *via* multiple hydrogen bonding and stacking (Zhou et al., 2012). Meanwhile, the internal pores are adjustable through the choice of appropriate monomers. Computational studies, X-ray diffraction, and microscopy results are in agreement with the construction of well-defined shaped-persistent nanotubes able to define stable nanopores.

The cyclic arylene-ethynylene macrocycle **4** (Figure 1) demonstrates excellent selective ion transport and high-water permeability. Its conductance reaches ~5.8 pS for K^+^ cations, while no transport is observed for Li^+^ or Na^+^ cations. The authors attribute this selectivity to the dehydration energy of cations based on MD simulations. Compound **4** also shows significant water permeability (2.6 ± 0.4 × 10^−14^ cm^3^ s^−1^, ~22% that of Aquaporin-AQP1) and single ion channel transport behaviors.

In addition, Kim and co-workers developed macrocyclic cucurbit[n]uril (CB[n]) derivatives as artificial ion channels (Jeon et al., 2004). By introduction of 3-octylsulfanylpropyl-O moieties into CB[n] (*n* = 6, 5), novel CB[n] derivatives effectively mediate the transmembrane proton/ion transport by a membrane mechanism. The results showed that the inherent cavity of the CB[n] play an important role in the transport of ions. With different hydrophobic cavities (CB[6], diameter ~5.5 A; CB[5], diameter ~4.4 A), the two CB[n]s show different ion selectivities. Based on the transport data and selectivity, the authors imply that CB[n] derivatives transport ions through their inherent cavities. Yet, the authors do not study the reconstitution of CB[6] derivatives in lipid bilayers and how they form ion channels. Notably, CB[6] derivative shows high ion flux of ~3^*^10^7^ ion/s, comparable to that of gA.

## Discussions

The aforementioned five artificial gA mimics demonstrate some similarities but also some differences. With respect to similarities, all of them were generated *via* self-assembly of molecular components through non-covalent interactions. All of the channels forming compounds are amphiphilic so that they present good partition into the lipid bilayers and facilitate the water/ion transporting. The absence of ion-exclusion sites causes them to form the non-exclusive channels but show many similarities with the natural gA. With respect to the differences, the first three channels **1**-**3** provide the hydrophilic pores for ion/water transport. The last two systems, **4** and CB[n] derivatives, offer a hydrophobic pore, able to provide a high flow velocity for ions and/or water owing to less friction like carbon nanotubes. The pores formed by **1** and **3** are chiral and contain oriented water wires in their confined space (Table 1).

**Table 1:** A summary of various artificial gA channels.

**Compound**	**Nature of the channel**	**Net permeability/selectivity/single channel permeability or ion flux**	**References**
**1**	Hydrophilic helical channel (2.8 Å) via π-π stacking of aromatic units	No permeability reported/high selectivity for water, reject all ions except protons	Zhao et al., 2014
**2a–2e**	Hydrophilic helical channel (1.0 nm) via intramolecular hydrogen bonding in aromatic hydrazide foldamers	No permeability reported/higher ${\text{NH}}_{4}^{+}$/K^+^ selectivity than that of gA	Xin et al., 2014
**3**	Self-assembled helical pores (~2.5–4 Å); double helical water channels with double helix net-dipolar orientation	No permeability reported for water/enhanced conduction states for alkali cations and for protons	Barboiu et al., 2015
**4**	Hydrophobic tubular channel (6.4 A) via tubular π-π stacking of macrocyclic arylene-ethynylenes and H-bonding of marginal dipeptides	51 μm s^−1^/no selectivity for water, high conduction for K^+^, and protons/4.9*10^7^ water molecules per s per channel	Zhou et al., 2012
**5**	Inherent hydrophobic cavity (~2.4–5.5 Å)	No permeability reported for water/enhanced conduction states for alkali cations and for protons/~3*10^7^ Cs^+^ ions per s per channel	Jeon et al., 2004

The behaviors of channels presented here may lead to more general conclusions. Structured water and derived physical theories have been the sources of continuous controversies. However, it should be stressed that moving from conventional bulk water to confined water is not simply a change of the scale, as the dynamics of confined water is quite fundamentally complex and different from that observed in bulk liquid water. Compartmentalization and chiral surfaces are basic features of biomolecules. The directional ordering of confined water-wires, as observed inside all presented gA mimics, is reminiscent with specific interactions between water and the biosurfaces, of which most are chiral. This dipolar orientation may induce important dielectric behaviors, which may certainly influence the biological recognition at biointerfaces or inside biocavities. The dipolar orientation of waters within ion-water single file through gA channel is an important driving force for the permeation of ions. The single-file columns of water are like a lubricant between the inner surface of the pore and the diffusing ions. On the other hand, such association of simple asymmetric properties: the *chirality of the pore structures* and one directional *orientation of the dipolar water-wires*, enables the idea for a novel strategy of much interest for artificial ion-pumping processes.

## Outlook

Nature has developed various ion/water channels over a million years or more. As an example of natural channels, gA has showed interesting and important functions, especially on water/ion transport. However, biomimetic on artificial ion/water channels has emerged in <4 decades, dating back to the seminal paper published in Tetrahedron Letters in 1982 by Tabushi et al. (1982) when Co^2+^ ions have been efficiently transported by a functionalized cyclodextrin carrier, with the rate 4.5 × 10^−4^ sec^−1^, through a bilayer membrane. Since this period, some artificial ion channels have surpassed gA in both cation/anion selectivity and proton/water transport efficiency, as well as active open channel-activity in bilayer membranes. It shows the bright future of research on artificial gA. It is still challenging for researchers to design the structures of artificial gA with expected selectivity and efficiency. The development of this field not only relies on further understanding on biology but also on organic synthetic strategies. With its progress, the applications of artificial gA will blossom.

## Author Contributions

ZS collected the references and wrote the draft. MB finalized the manuscript.

### Conflict of Interest Statement

The authors declare that the research was conducted in the absence of any commercial or financial relationships that could be construed as a potential conflict of interest.
